# Ethnobotanical Survey and Cercaricidal Activity Screening of Medicinal Plants Used for Schistosomiasis Treatment in Atwima-Nwabiagya District, Ashanti Region, Ghana

**DOI:** 10.1155/2023/6707157

**Published:** 2023-07-19

**Authors:** Evelyn Asante-Kwatia, Lord Gyimah, Arnold Donkor Forkuo, William Kofi Anyan, Makafui Adzo Gbemu, Francis Ackah Armah, Abraham Yeboah Mensah

**Affiliations:** ^1^Department of Pharmacognosy, Faculty of Pharmacy and Pharmaceutical Sciences, College of Health Sciences, Kwame Nkrumah University of Science and Technology, Kumasi, Ghana; ^2^Department of Pharmacology, Faculty of Pharmacy and Pharmaceutical Sciences, College of Health Sciences, Kwame Nkrumah University of Science and Technology, Kumasi, Ghana; ^3^Noguchi Memorial Institute for Medical Research, College of Health Sciences, University of Ghana, Legon, Ghana; ^4^Department of Biomedical Sciences, School of Allied Health Sciences, College of Health and Allied Science, University of Cape Coast, Cape Coast, Ghana

## Abstract

This study focused on documenting and evaluating the cercaricidal activity of medicinal plants used for schistosomiasis treatment in an endemic area in Ghana. Through semistructured questionnaires, personal interviews with herbalists in communities surrounding the Barekese dam in the Atwima-Nwabiagya district, where the disease is endemic, were carried out. Thirty medicinal plants distributed in 19 families were reported to be used for schistosomiasis treatment in the survey. Information on the plants, including scientific names, common names, families, and the used plant part were recorded. The families Apocynaceae and Euphorbiaceae recorded the highest number of plants (14% each), followed by Asteraceae (10%), Loranthaceae (7%), and Rubiaceae (7%). *In vitro* cercaricidal activity of methanol extracts of nine out of the thirty plants was performed by exposing human *Schistosoma mansoni* cercariae obtained from *Biomphalaria pfeifferi* to various concentrations of extracts over a duration of 240 minutes. All the plants tested demonstrated time- and concentration-dependent cercaricidal activity. With lethality being set at <1000 *μ*g/mL, the cercaricidal activity in order of decreasing potency was as follows: *Withania somnifera* (LC_50_ = 1.29) > *Balanites aegyptiaca* (LC_50_ = 7.1) > *Xylia evansii* (LC_50_ = 11.14) *> Jathropha multifida* (LC_50_ = 12.9) *> Justicia flava* (LC_50_ = 22.9) *> Anopyxis klaineana* (LC_50_ = 182.81) > *Ximenia americana* (LC_50_ = 194.98) *> Loranthus lecardii* (LC_50_ = 223.87) *> Bridelia tenufolia* (LC_50_ = 309.03) > *Zanthoxylium zanthoxyloides* (LC_50_ = 851.94). Phytochemicals, including alkaloids, tannins, triterpenes, saponins, phytosterols, and flavonoids were identified in the plants. The result of this study gives scientific credence to the traditional use of these plants in the treatment of schistosomiasis and proves that the rich botanical knowledge of medicinal plants provides an incredible starting point for the discovery of new anti-schistosomal drugs for the local population.

## 1. Introduction

Schistosomiasis is the second most endemic parasitic worm disease caused by species of dioecious blood flukes of the genus *Schistosoma*, including *Schistosoma guineensis*, *Schistosoma haematobium*, *Schistosoma intercalatum*, *Schistosoma japonicum*, *Schistosoma mansoni*, *and Schistosoma mekongi* [1]. More than 200 million individuals in tropical and subtropical regions of the world are affected by this neglected tropical disease; especially in deprived communities, which lack access to safe drinking water and adequate sanitation [2]. High prevalence rates have been recorded in Africa, the Caribbean, Latin America, and Asia with over 90% of cases reported in sub-Saharan Africa [3].

Considerable efforts to eradicate schistosomiasis from endemic areas through repeated mass preventive chemotherapy, improvement of water, sanitation, and hygiene, snail control, behavioural changes, and environmental management have not been entirely successful [4]. This is mainly due to the high cost involved in treatment campaigns (including basic health education, drugs, and logistics) and the inequity of access to preventive chemotherapy [5, 6]. Moreover, praziquantel, which is currently the only drug of choice for treatment, has been faced with the challenge of reduced drug sensitivity to juvenile parasites, and the suspected emergence of drug-resistant strains [7–9]. While emphasizing the need to eliminate schistosomiasis by 2030, the World Health Organization (WHO) proposed the development of new intervention tools and alternative drugs to praziquantel [10].

In Ghana, schistosomiasis is present in all sixteen regions, mostly in rural and some rural-urban communities [11]. Even though mass drug administration began in 2008 and has continued, the disease is still highly endemic with a nationwide prevalence rate of ~23% reported in 2015 and a focal prevalence of >50% in the country's capital [12, 13]. Between 2017 and 2021, the population in need of preventive chemotherapy for schistosomiasis in Ghana increased from 10.1 to 11.5 million with only 24.1% receiving preventive chemotherapy [14]. High disease prevalence has been reported from communities around the Densu River in Greater Accra and Eastern regions [13, 15], towns surrounding the Barekese and Owabi dams in the Ashanti region [16], along the Tono irrigation dam in Northern Ghana [17], and along the volta basin in the Volta region [18]. The construction of dams, lack of potable water, and dependence of the riparian communities on river sources are some reasons for this high prevalence in these districts.

As is the case in most African countries, many people resort to the use of traditional herbal remedies for the treatment of schistosomiasis in Ghana. This is mainly due to their affordability, accessibility, efficacy, and perceived safety over orthodox medicines [19, 20]. As usual, the efficacy claims for most of these herbal remedies are based on past experiences without any scientific proof. In recognizing the importance of medicinal plants in the health care system of developing countries, the WHO encouraged the inclusion of herbal medicines with sufficient data on efficacy and safety into mainstream clinical practice [21]. Given this, the 21st century witnessed several research reports from the African Continent, focusing on the validation of the efficacy of traditionally used anti-schistosomal plants [21–25]. The artemisinin derivatives, artesunate and artemether from *Artemesia annua*, and myrrh from *Commiphora momor* are examples of plant-based products that are currently registered for the treatment of schistosomiasis in some African countries [26, 27].

The Ghanaian flora is an invaluable source of new medicinal agents for the local population [28]. Unfortunately, the significance of these medicinal plants in the bioeconomy has so far not been realized. Traditional medical approaches to the treatment of schistosomiasis in Ghana are not well documented, and only a few studies exist on the anti-schistosomal activities of Ghanaian medicinal plants [21, 29, 30]. This research was thus undertaken to document herbal remedies used to treat schistosomiasis in selected communities surrounding the Barekese dam in the Atwima-Nwabiagya district, where the disease was reported to be endemic [16]. Consequently, knowledge of such herbal medicines, followed by biological activity testing, could lead to the identification of potential anti-schistosomal remedies for the future development of affordable community-based remedies for incorporation into primary health care.

## 2. Materials and Methods

### 2.1. Chemicals

Analytical grade organic methanol used for extraction was obtained from BDH Laboratory elements (Merck Ltd., Lutterworth, UK). All other chemicals used in the various experiments were purchased from Sigma–Aldrich Co. Ltd. Irvine, UK.

### 2.2. Ethical Clearance

To participate in this survey, participants gave their voluntary oral or written prior informed consent through the Committee on Human Research Publication and Ethics (CHRPE), KNUST Participant Consent Form. No further ethics approval was required.

### 2.3. Study Area

**Figure 1:**
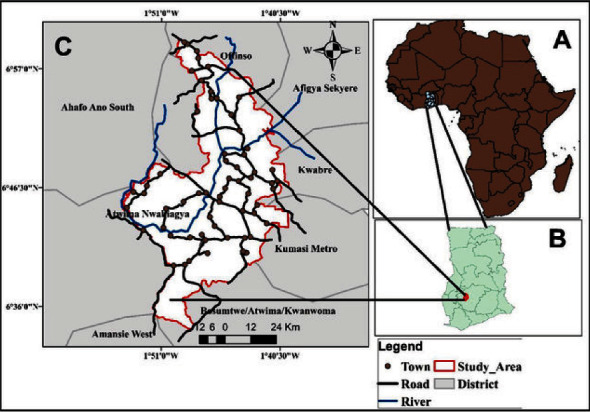
Map of Atwima-Nwabiagya district (*Source*: Adapted from Ref. [32]).

The present study was carried out in the ‘Atwima-Nwabiagya' district (latitude 6°40′0″N and longitude 1°49′0″W) in the Ashanti region of Ghana (Figure 1). It is 1 of the 21 political and administrative districts in the Ashanti region [31]. According to the 2010 Population and Housing Census in Ghana [63], the district covers an estimated area of 2,411 km^2^ and an average height of about 77 m above sea level. The population of the district is about 149,025 with the majority (68.5%) of the population living in rural localities (2010 Population and Housing Census, Ghana) [63]. The surface area of the district is mainly drained by the ‘*Offin'*, ‘*Owabi'*, and ‘*Tano'* rivers. There are, however, several streams in the district. These include ‘*Kobi'* and ‘*Dwehen'*. ‘*Owabi'* and ‘*Barekese'* are the two major dams have been constructed across the ‘*Owabi'* and the ‘*Offin'* rivers, respectively (2010 Population and Housing Census, Ghana) [63]. The major occupation in the district is wholesale and retail trade, cocoa farming, forestry, and fishing [32]. The Atwima-Nwabiagya district was selected for this survey based on the previously reported prevalence of urinary schistosomiasis in the area [16].

### 2.4. Sampling Procedure and Data Collection Methods

The ethnobotanical survey was carried out in August 2020 in Marbang, Adankwame, Ayensua fufuo, Ayensua kokoo, Barekese, Barekuma, and Akyena, which are all rural to urban-rural settlements through which tributaries of the Offin River are found. Eleven herbalists in the community were identified with assistance from community members and interviewed one on one using open-ended semistructured pretested questionnaires, designed in English and translated into the local dialect, Asante-Twi. Information on biodata, knowledge of schistosomiasis, and plants used to treat schistosomiasis were documented.

### 2.5. Harvesting and Collection of Plant Materials

A review of the literature on the documented plants (Table 1) revealed that some had been previously investigated for anti-schistosomal activity. Based on this report, nine out of the thirty listed plants were selected for cercaricidal activity screening. The selected medicinal plant materials were harvested in November 2020 from various localities in the survey area. The specific plant parts harvested are listed in Table 2. The plants were identified and authenticated by Dr. George Henry Sam of the Herbal Medicine Medicine Department, Faculty of Pharmacy and Pharmaceuticals Sciences, KNUST. Voucher specimens were generated and deposited at the Department of Herbal Medicine's Herbarium.

### 2.6. Processing and Preparation of Plant Extracts

The freshly collected plant materials were washed under running water to do away with any foreign matter present. The plant parts harvested are listed in Table 2. They were then chopped into smaller pieces, air-dried at room temperature for two weeks, and mechanically ground into a coarse powder. Various quantities of the powdered samples of the selected plants were weighed separately and cold-macerated with methanol for 48 hours. The extracts derived were concentrated with the aid of a rotary evaporator (BUCHI Rotavapor, Flawil, Switzerland) under reduced pressure at 65°C and further evaporated to dryness in an oven (electronic Gallenkamp) at 40°C. The extracts were kept in air-tight containers in a refrigerator for further work.

### 2.7. Preliminary Phytochemical Screening

The dried powdered plant material of the plants was screened for plant secondary metabolites, such as glycosides, tannins, phenolic compounds, alkaloids, sterols, flavonoids, and terpenoids, following previously established methods [33].

### 2.8. Biological Activity Studies

#### 2.8.1. Materials and Equipment

The materials and equipment used are as follows: aquarium plastic bowl (measuring 27 cm × 12 cm), aquarium net/sieve, aged water, beakers, artist brush, fresh lettuce, forceps, distilled water, 24-microtiter plate, Pasteur pipette, inverted microscope (IV950 series), tally counter, vortex, and vacuum tube.

#### 2.8.2. Harvesting of the Intermediate Host Snails

Harvesting of the intermediate host snails, *Biomphalaria pfeifferi*, was carried out following the method of Abe et al., and the method description partly reproduces their wording [34]. Briefly, fresh water host snails of *S. mansoni* parasites were scouted for and collected from their natural habitat in River Densu at Tomefa near Weija in the Ga South Municipal District, Greater Accra Region of Ghana. Harvesting was done by examining different sites of the river where they normally reside, thus, water, plants, and muddy areas that are rich in decaying organic matter, rocks, and stones. With the aid of forceps and an artist's brush, the fresh water snails were collected into aquarium plastic bowls partially filled with fresh water to mimic their natural habitat. The collected snails were first washed with fresh water to remove debris and snail excreta with an aquarium net/sieve, then subsequently washed with aged water (tap water that has been kept in a container and covered for 14 days). They were kept in beakers half-filled with aged water and transported in well-ventilated aquarium plastic bowls (measuring 27 cm × 12 cm) to the Parasitology Laboratory at Noguchi Memorial Institute for Medical Research (NMIMR), Accra, where the experiment was performed.

#### 2.8.3. Maintenance of Host Snails

The transported snails were maintained in the Parasitology Laboratory at NMIMR under standard laboratory conditions at a temperature of 28°C. The snails were fed with fresh lettuce and left to acclimatize to the laboratory conditions overnight. The water in the aquarium was changed every day until the work was completed.

#### 2.8.4. Exposure of Host Snails and Cercariae Shedding

Cercariae shedding was accomplished following the method by Obare et al., and the method description partly reproduces their wording [35]. Briefly, single snails were individually placed in each well of a 24-well microtiter plate containing 1 mL of distilled water with the aid of forceps. The plates were covered to prevent snails from crawling out and were placed under an artificial lamp (fluorescent bulb 60 W) for an hour. After an hour, the snails were observed for shedding human *S. mansoni* cercariae under an inverted microscope (IV950 series) at ×4 objective lens magnification. Snails with positive cercariae shedding ability were separated and maintained accordingly.

#### 2.8.5. Standardization of Cercariae Solution

Cercariae suspensions were transferred into 50 mL vacuum tubes with the aid of slantly cut micropipettes to prevent the cercariae from distortion or breaking their head or tail. An aliquot of 50 *μ*L parasite suspension was spread onto a glass slide and observed under a light microscope (Leica Light Microscope DM 1000 LED, Wetzlar, Germany) to estimate the number of cercariae per 50 *μ*L. The assay was performed in triplicates. From the estimated number of cercariae per microlitre, the cercariae stock suspension was diluted with distilled water such that each well of a 24-well microtiter plate will contain approximately 15 ± 3 cercariae per 500 *μ*L.

#### 2.8.6. Cercaricidal Activity of Plant Extracts

Stock extract concentrations of 1000 *μ*g/mL were prepared from the methanol extracts of plants. Serial dilution (twofold) of the stock solution with distilled water was performed to obtain concentrations of 500.0, 250.0, 125.0, 62.5, 31.25, and 15.625 *μ*g/mL. The standard drug for schistosomiasis treatment, praziquantel is active against adult schistosoma worms, whereas immature stages, including cercariae are largely insensitive to this drug [36, 37]. Hence, praziquantel was not employed as a positive control in cercaricidal activity screening. The plant *Balanites aegyptiaca* is reported to exhibit significant cercaricidal activity against *S. mansoni* cercariae [38–40] and has been employed as an internal control to evaluate the adequacy of experimental conditions in many studies evaluating the cercaricidal activity of plants [29, 41]. Hence *B. aegyptiaca* extracts, prepared at the same concentrations of test samples, were employed as an internal control, and distilled water was used as the negative control in this study. The cercaricidal activity was performed according to the method described by Tekwu et al., and the method description partly reproduces their wording [42]. Briefly, an aliquot of 500 *μ*L of parasite stock (containing approximately 15 freshly shed cercariae) was dispensed in each well of a 24-microtiter plate using a micropipette. An aliquot of 500 *μ*L of the extract dilution was added to the parasite suspension. The wells were then observed for cercaricidal activity using an inverted light microscope (Optika IM-3LD2 Inverted LED, 400×, Germany) under ×4 magnification for 4 hours at time intervals: 15, 30, 60, 90,120, 150, 180, 210, and 240 minutes. Morphological changes of cercariae and the time of cercariae death were recorded. Cercariae was presumed dead when they were immobile, lost their tails or head and/or coiled body, and sank to the bottom of the well. The number of live cercariae at the beginning of the experiment and the number of live cercariae at the observation time were recorded. All experiments were carried out in triplicates. The LC_50_ values of the plant extracts against *S. mansoni* cercariae were determined. The percentage mortality was calculated as;

(1) $$\begin{array}{c} \text{Percentage mortality}=\frac{\text{Initial count of live cercariae}-\text{Final count of live cercariae}}{\text{Initial count of live cercariae}}\times 100\%. \end{array}$$

### 2.9. Statistical Analysis

All data were calculated as the mean ± standard error of the mean (SEM). Statistical analysis was carried out using the Graphpad^®^ Prism Version 7.0 (Graphpad Software, San Diego, CA, USA) for Windows. Time-course curve of the percentage mortality of the plant extracts against time was plotted. The LC_50_ (i.e., the concentration at which 50% of the cercariae died was determined by plotting a nonlinear regression curve (log concentration of inhibitor vs. % mortality).

## 3. Results

### 3.1. Ethnobotanical Studies

#### 3.1.1. Socio-Demographic Details of Respondents

Eleven traditional medicine practitioners, who were identified by community members in the study area were interviewed during the survey. All traditional healers interviewed belonged to the Akan tribe. The inclusion criterion for the survey was that the practitioner used herbal medicine. They comprised 77% men and 23% women aged between 31 and 70 years. A majority of respondents (61%) had practiced for more than 15 years.

#### 3.1.2. Knowledge and Perception of Healers about Schistosomiasis

All interviewed herbalists reported having heard of and knew urinary schistosomiasis, which they locally referred to as *‘gyonso mogya'*, literally meaning ‘bloody urine' in the Akan language, alluding to haematuria, which was the most common symptom of the disease presentation. Meanwhile, none of the respondents knew about any other form of the disease when asked about intestinal schistosomiasis. They all responded affirmatively to treating at least one patient with ‘bloody urine' in the past one year.

Even though the herbalists interviewed reported treating only urinary schistosomiasis (or its major symptom-haematuria), interactions with community members within the same locality, indicated intestinal symptoms, such as bloody stool, diarrhoea, and abdominal pain (*unpublished results*). From the responses from herbalists and community members, it could be inferred that intestinal schistosomiasis may not be uncommon in the area visited. Its symptoms may have been misconceived for other gastrointestinal tract infections. It was expected that nearby water bodies could be a source of the intermediate hosts for both *S. haematobium* and *S. mansonii*. However, only few *B. pfeifferi* were obtained from nearby river sources. This led to the selection of this species for the study.

Despite the appreciable level of awareness of the disease in the area, correct knowledge about the specific cause and modes of transmission was not adequate among respondents. In response to a multiple-choice question on the cause of schistosomiasis, more than half of the herbalists mentioned ‘infected water' (68.8%) and ‘some types of worms' (77.9%) as causes of the disease, whereas about 38% associated the disease with personal hygiene. The most frequently mentioned mode of transmission related to contact with infected water (72.1%) or ‘bite' from a fresh water snail (32.2%), even though some respondents still believed that sex with infected persons (3.8%) or sharing toilet facilities with infected persons (15.5%) could also be a transmission route for the disease. Knowledge of the symptoms of schistosomiasis varied, with most of the respondents listing blood in urine, painful urination, and other non-specific symptoms, such as fever, vomiting, dizziness, and loss of appetite.

#### 3.1.3. Medicinal Plants Used in the Treatment of Schistosomiasis

**Figure 2:**
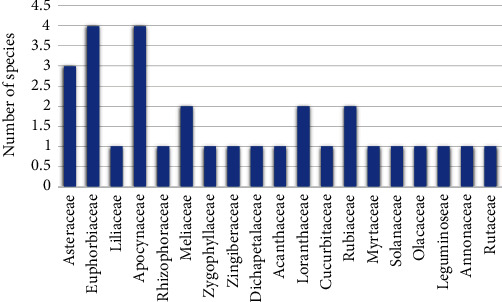
Families of anti-schistosomal plants recorded in Atwima-Nwabiagya District, Ghana.

**Figure 3:**
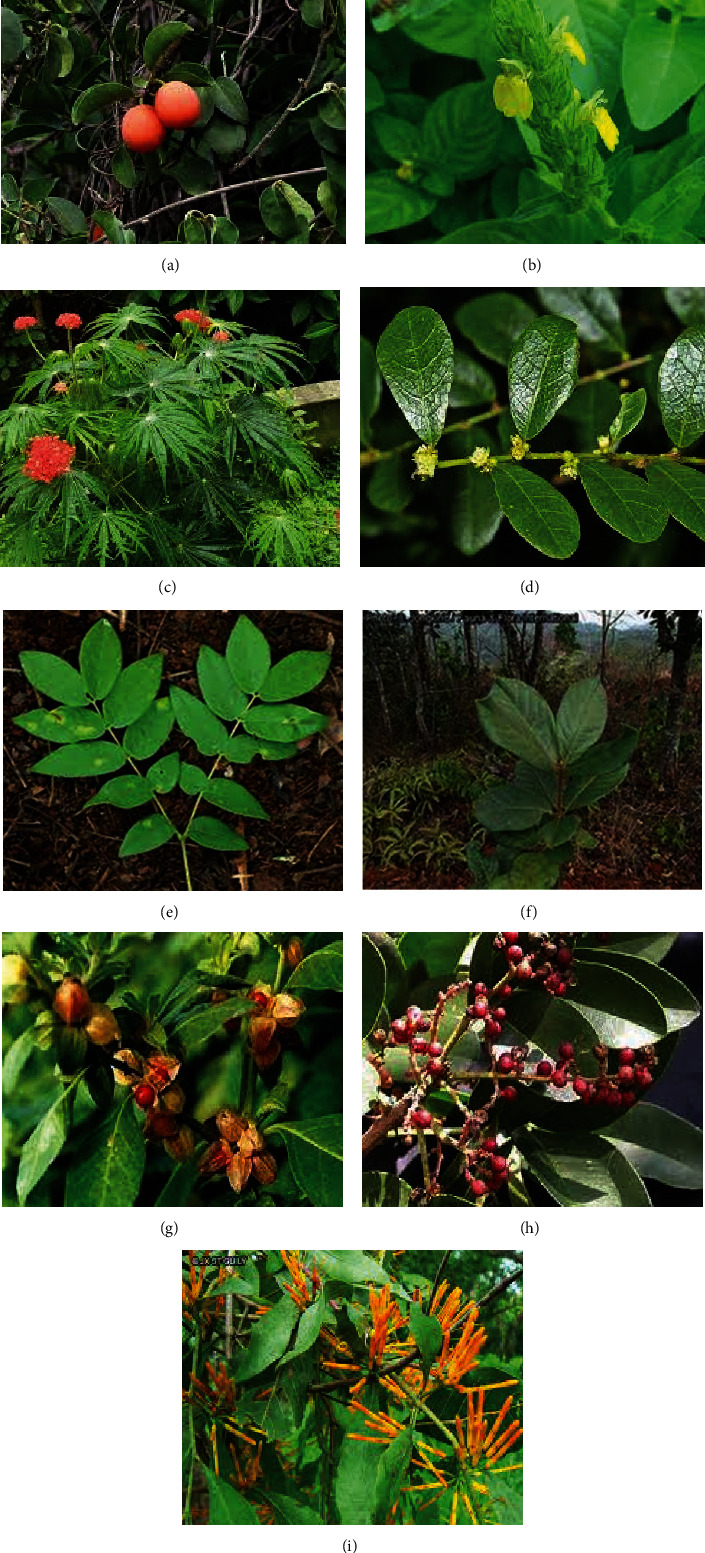
Selected plants for cercaricidal activity screening. (a) *X. americana*. (b) *J. flava*. (c) *J. multifida*. (d) *B. tenufolia*. (e) *X. evansii*. (f) *A. klaineana*. (g) *W. somnifera*. (h) *Z. zanthoxyloides*. (i) *L. lecardii*.

A total of 30 medicinal plants distributed in 19 families were cited by the herbalists to be used in the treatment of schistosomiasis. The families Apocynaceae and Euphorbiaceae recorded the highest number of plants (14% each), followed by Asteraceae (10%), Loranthaceae (7%), and Rubiaceae (7%). Other plant families, including Dichapetalaceae, Olacaceae, Acanthaceae, Phyllanthaceae, Leguminoceae, Rhizophoraceae, Solanaceae, Zygophallaceae, Rutaceae, Liliaceae, Meliaceae, Cucurbitaceae, Myrtaceae, and Annonaceae recorded 3% each (Figure 2). Data from the survey comprising of the plants' scientific names, families, common names, growth habitats, and used part(s) are presented in Table 1. Based on existing reports on the anti-schistosomal effect of the listed plants and the availability of samples, nine plants, namely: *Ximenia americana*, *Justicia flava*, *Jathropha multifida*, *Bridelia tenufolia*, *Xylia evansii*, *Anopyxis klaineana*, *Withania somnifera*, *Zanthoxylum zanthoxyliodes*, and *Loranthus lecardii* were selected for cercaricidal activity screening (Figure 3).

#### 3.1.4. Phytochemical Screening of Selected Plants

The results of the phytochemical analysis of selected medicinal plants are summarized in Table 2.

### 3.2. Cercaricidal Activity of Selected Plants

#### 3.2.1. General Effect of the Extracts on *S. mansoni* Cercariae

**Figure 4:**
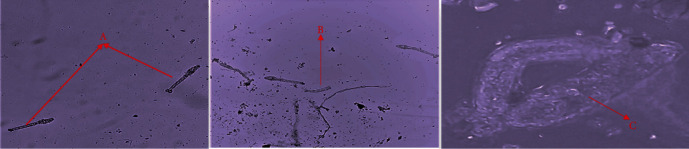
Cercariae behaviour before and after treatment with plant extract. (A) Intact cercariae with head and tail before treatment (×10). (B) Loss of head in cercariae (×10). (C) Coiling of cercariae (×40).

Cercariae were very active and mobile at the initial stage of the experiment before exposure to the plant extracts. After exposing the cercariae to various concentrations of the extracts, the cercariae became sluggish, inactive, and/or immobile. Furthermore, morphological/tegumental changes, such as loss of bifocal tail, loss of head (head separation from the tail), abnormal shape/coiling up, and change in colour were observed at some concentrations (Figure 4).

#### 3.2.2. Cercaricidal Activity of the Plant Extracts

**Figure 5:**
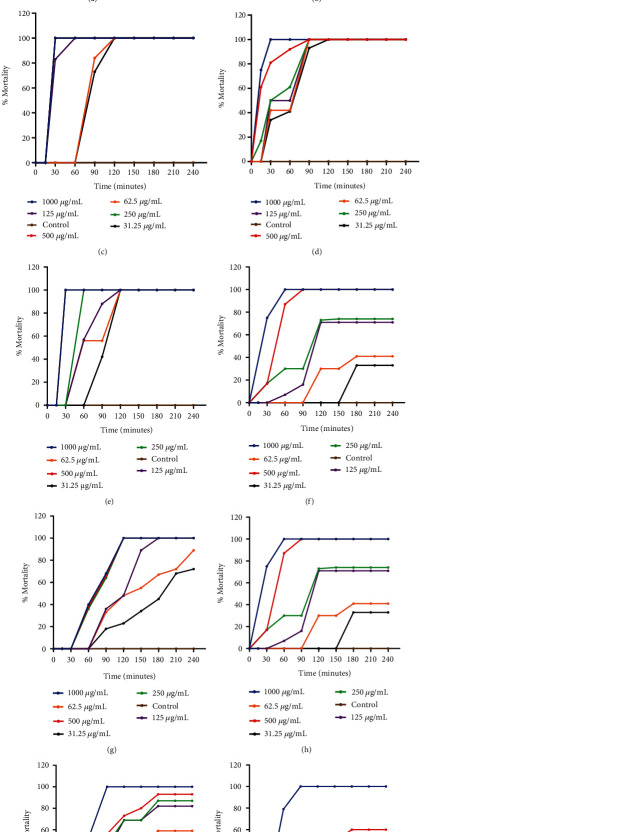
Time-course curves of cercariae mortality after treatment with plant extracts. (a) *W. somnifera*. (b) *B. aegyptiaca*. (c) *X. evansii*. (d) *J. multifidai*. (e) *J. flava*. (f) *A. klaineana*. (g) *X. americana*. (h) *L. lecardii*. (i) *B. tenufolia*. (j) *Z. zanthoxyloides*.

All extracts exhibited a concentration and time-dependent cercaricidal activity against *S. mansoni* cercariae. The time-course curves for all extracts are presented in Figures 5(a), 5(b), 5(c), 5(d), 5(e), 5(f), 5(g), 5(h), 5(i), and 5(j). The most effective extracts were *W. somnifera*, *X. evansii*, *J. multifida*, and *J. flava*, which caused an average percentage mortality of >70% by the 15th minute and attained 100% mortality between the 15th and 60th minutes for 1,000 *μ*g/mL extract (Figures 5(a), 5(b), 5(c), and 5(d)). For *J. multifida* and *W. somnifera* extracts, concentrations ranging between 62.5 and 500 *μ*g/mL attained 100% mortality not later than the 90th minute, whereas at 31.25 *μ*g/mL, attained 100% mortality at the 120th minute (Figures 5(a) and 5(b)). For the standard control, *B. aegyptiaca* concentrations of 500–1000 *μ*g/mL attained ~40% to 50% mortality by the 15th minute, and 100% mortality at the 60th minute. At 125–250 *μ*g/mL, *B. aegyptiaca* attained 100% mortality by the 90th minute, whereas concentrations of 31.25–62.5 *μ*g/mL recorded 100% mortality at the 120th minute (Figure 5(j)). For the other plants screened, that is, *B. tenufolia*, *Z. zanthoxyloides*, *L. lecardi*, and *X. americana*, 1,000 *μ*g/mL caused 100% mortality not earlier than the 90th minute and concentrations ranging between 31.25 and 500 *μ*g/mL attained 100% mortality between the 90th and 240th minutes (Figures 5(f), 5(g), 5(h), and 5(i)).

The least concentration of the extract that caused 50% cercariae mortality (LC_50_) was determined from a plot of % mortality against log concentration for all extracts. From this analysis, the standard control, *B. aegyptiaca* had an LC_50_ of 7.1 with a minimal lethal concentration (MLC) of 31.25 *μ*g/mL, achieving 100% mortality at 120 minutes. *W. somnifera* exhibited a much more potent cercaricidal activity with an LC_50_ of 1.29 and an MLC of 31.25 *μ*g/mL, achieving 100% mortality by the 120th minute. This was followed by *X. evansii* (LC_50_ = 11.14) and *J. multifida* (LC_50_ = 12.90), both having an MLC of 31.25 *μ*g/mL, achieving 100% mortality by the 120th minute. Among all extracts tested, *B. tenufolia* and *Z. zanthoxyloides* exhibited the least cercaricidal activity having LC_50_ values of 309.03 and 851.94, respectively, with MLCs of 1,000 *μ*g/mL for both extracts (Tables 3 and 4).

## 4. Discussion

The high incidence of schistosomiasis in some Ghanaian communities, coupled with the unavailability and reduced efficacy of praziquantel, the drug of choice, has prompted the exploration of other sources of new or alternative treatment options for the disease. In several African countries, including Ghana, medicinal plants have proven to be an invaluable source of healing agents for the treatment of a plethora of diseases. This rich botanical knowledge provides an incredible starting point for the discovery of new drugs for the local population. In this study, medicinal plants used in the treatment of schistosomiasis in selected endemic communities around the Barekese Lake in the Atwima-Nwabiagya District of Ghana were recorded in an ethnobotanical survey. Furthermore, the cercaricidal activity of selected plants was investigated against the *S. mansoni* cercariae in an *in vitro* assay.

From the ethnobotanical survey, thirty medicinal plants belonging to 19 families were listed by herbalists to be used by them in the management of schistosomiasis. According to the practitioners, formulations comprised of one to three plants dispensed as dried powdered herbs are to be prepared as decoctions for oral administration for at most 2 weeks. The efficacy claims were based on the number of people who experienced reduction or relief from symptoms as a result of using the herbal formulations. The use of medicinal plants for the treatment of schistosomiasis is a common practice in several African countries. Previous ethnopharmacological studies make mention of the use of different plants in the treatment of schistosomiasis in South Africa [44], Zimbabwe [45], Mali [46], Kenya [25], Morocco [47], and Ivory Coast [48]. Some of the plant species listed in the ethnobotanical survey conducted had been previously reported in other studies for cercaricidal and anti-schistosomal activity. In a study by Acheampong et al., *Azazdirachta indica* and *Nauclea latifolia* exhibited a very potent cercaricidal effect on *S. mansoni* cercariae, and further significantly reduced worm burden in schistosomiasis-infected mice [29]. In another study, solvent fractions and erythroivorensin, a flavanone isolated from the roots of *Erythrophluem ivorense*, displayed a remarkable cercaricidal effect against *S. haematobium* cercariae [30]. The stems and roots of *D. crassifolium* together with its triterpenoids and sterol isolates also demonstrated significant cercaricidal activity against *S. haematobium* [43]. In a recent study, the hydroethanolic and alkaloidal extracts from the stem bark of *H. floribunda* also showed potent cercaricidal activity against *S. haematobium* cercariae [41]. Others include preparations from *Khaya senegalensis*, *V. amygdalina*, *A. indica*, *A. barbadensis*, and *T. officinale*, which exhibited varying inhibitory effects against newly transformed schistomula [21]. These scientific reports, justifying the traditional claims of these plants in the treatment of schistosomiasis, confirm the important relationship between therapeutic agents derived from plants and their ethnomedicinal applications.

Nine out of the thirty listed medicinal plants were tested for cercaricidal activity against *S. mansoni*. All plants tested demonstrated time- and concentration-dependent cercaricidal activity against *S. mansoni* cercariae at varying levels. This was congruent with previous studies on the cercaricidal effects of plant extracts, which also showed a time and concentration relationship for various extracts against *Schistosoma* parasites [42]. Probit analysis of all plants' activities showed a strong positive correlation between plant concentration and mortality. As established earlier, *B. aegyptiaca*, used as the standard control, was reported to exhibit very significant activity against *Schistosoma* cercariae [29]. The cercaricidal activity of the plants screened in order of decreasing potency (LC_50_) was as follows: *W. somnifera* > *B. aegyptiaca* > *X. evansii > J. multifida > J. flava > A. klaineana* > *X. americana > L. lecardii > B. tenufolia* > *Z. zanthoxyloides.*

*W. somnifera* showed the highest cercaricidal activity against *S. mansonii* cercariae with an LC_50_ of 1.29. Similar to *B. aegyptiaca*, *W. somnifera* had a MLC of 31.25 *μ*g/mL at 120 minutes. *W. somnifera* is widely used in folk medicine in the treatment of several diseases. Its medicinal properties have been mainly attributed to the presence of steroidal lactones called withanolides, which have shown antiparasitic, antibacterial, antiviral, molluscicidal, anthelminthic, and immune-modulating potencies [49, 50]. In previous studies, formulations of *W. somnifera* leaves were found to be toxic to intermediate host snails of *Schistosoma haematobium*, that is, *Biomphalaria alexandrina* and *Bulinus truncatus*, through the destruction of their nucleic acids, proteins, and fatty acids, limiting their spread and reproduction rates [51]. Extracts from the leaves, fruits, and roots as well as isolated withanolides also demonstrated significant inhibitory effects against other human parasites including the leishmania parasite, *Leishmania donovani* [52, 53] and filarial parasite, *Brugia malayi* [54]. The significant cercaricidal activity exhibited by *W. somnifera* in this study, together with its previously reported antiparasitic activity, connotes its potential as a novel source of the drug candidate for the treatment of schistosomiasis.

*J. multifida* exhibited remarkable cercaricidal activity with an LC_50_ of 12.9 and MLC at 31.25 *μ*g/mL within 120 minutes. Previous studies reported on the cercaricidal activity of some *Jathropha* species including *Jathropha curcas* and *Jathropha elliptica* against *S. mansoni* cercariae. In other reports, some species of the genus *Jathropha*, including *Jathropha gossypiifolia*, *J. curcus*, and *J. elliptica* showed highly toxic effects on the *Schistosoma* vector snails, *Biomphalaria glabrata*, *B. truncatus*, and *Bulinus natalensis* [55]. This implies that extracts and oils from *Jathropha* species may be useful as both vector control and treatment agents in schistosomiasis-endemic regions. Diterpenes and pyridine alkaloids identified in these species were responsible for the inhibition of oviposition in *S. mansoni*, causing external and internal damage to structures in adult worms [56, 57]. The current result on the cercaricidal activity of *J. multifida* is congruent with these previous observations and suggests that *Jathropha* species, in general, may have a potential inhibitory effect on *Schistosoma* parasites and intermediate host snails.

This is the first report on anti-schistosomal activity of the genus *Xylia*. *X. evansii* exhibited the second most potent cercaricidal activity with an LC_50_ of 11.14 and MLC at 31.25 *μ*g/mL within 120 minutes.

The cercaricidal activity demonstrated by the plants under study could be attributed to their secondary metabolites, such as flavonoids, saponins, and alkaloids [58]. In previous studies, the total alkaloids, saponins, and essential oils from *Nigella sativa* [59], isoflavonoids from *Millettia thonningii* [60], and alkaloids from *J. elliptica* [57] demonstrated significant cercaricidal activity against *S. mansoni* cercariae. Plant essential oils have also been reported to induce catalytic activity of acetylcholinesterase (AChE), which damages the cholinergic nervous system of cercariae. This interferes with cercariae motor activity leading to atypical movements, such as slow rotation and vibration as well as contortions [61]. These reports on cercaricidal activity of plant phytoconstituents, strongly support the effect of some of the plants under study in killing *S. mansoni* cercariae. According to Xiao et al. [62], cercaricidal activity usually results from an intensive disturbance in motor activity and lysis of cercarial tissues, followed by an extensive release of gland contents and separation of the tail from the body. Specifically, in this study, separation of cercariae head and tail as well as cercariae coiling was observed.

## 5. Conclusion

This study has highlighted the importance of medicinal plants in the treatment of schistosomiasis in Ghanaian communities. Furthermore, the remarkable cercaricidal activity demonstrated by some plant species provides scientific credence for their ethnomedicinal use and paves the way for further investigation into their anti-schistosomal activity.

## Figures and Tables

**Table 1:** Medicinal plants used to treat schistosomiasis in Atwima-Nwabiagya, Ghana.

Scientific name	Family	Common name	Life form	Part used	References
*Ageratum conyzoides* Linn.	Asteraceae	Goat weed	Herb	Leaves and whole plant	[21]
*Alchornea cordifolia* Müll. Arg.	Euphorbiaceae	Christmas bush	Shrub	Leaves	[21]
*Aloe barbadensis* Miller	Liliaceae	Aloe vera	Shrub	Leaves	[21]
*Alstonia boonei* De wild	Apocynaceae	Alstonia	Tree	Leaves and stem bark	[21]
*A. klaineana* Pierre Engl.	Rhizophoraceae	White oak/*kokoti*	Tree	Stem bark	—
*Azadirachta indica* A. Juss.	Meliaceae	Neem	Tree	Leaves	[29]
*B. aegyptiaca* L. Del	Zygophyllaceae	Soap berry	Tree	Seeds	[29]
*B. tenufolia* Müll. Arg.	Zingiberaceae	—	Shrub	Stem bark	—
*Dichapetalum crassifolium* Chodat	Dichapetalaceae	—	Shrub/climber	Roots and leaves	[43]
*Erythrophleum ivorense* Afzel.	Euphorbiaceae	Ordeal tree	Tree	Leaves and stem bark	[30]
*Holarrhena floribunda* (G. Don) Dur. & Schinz.	Apocynaceae	False rubber tree	Tree	Stem bark	[41]
*J. multifida* Linn.	Euphorbiaceae	Coral plant	Shrub	Stem bark	—
*J. flava* Vahl.	Acanthaceae	Afama	Herb	Inflorescence and leaves	—
*Kyaya senegalensis* (Desr.) A. Juss.	Meliaceae	African mahogany	Tree	Stem bark and leaves	[21]
*L. lecardii* Engl.	Loranthaceae	—	Shrub	Stem bark	—
*Momordica charantia* Linn.	Cucurbitaceae	Bitter gourd	Climbing herb	Whole plant	[21]
*Morinda lucida.* Benth.	Rubiaceae	Brimstone tree	Shrub	Leaves	[29]
*Nauclea latifolia* smith.	Rubiaceae	African peach	Shrub	Leaves	[29]
*Phyllanthus amarus* Schum. and Thonn.	Euphorbiaceae	Carry me seed	Herb	Whole plant	[29]
*Picralima nitida* Stapf T. Durand	Apocynaceae	*Akuama*	Tree	Seeds	[21]
*Rauwolfia vomitoria* Afzel.	Apocynaceae	Snake root	Shrub	Roots and stem bark	[29]
*Syzygium aromaticum* L. Merr	Myrtaceae	Clove	Shrub	Fruits	[21]
*Tapinanthus bangwensis* (Engl.& K. K.) Danser	Loranthaceae	African mistletoe	Shrub	Whole plant	[21]
*Taraxacum officinale* L. Weber	Asteraceae	Dandelion	Herb	Leaves	[21]
*Vernonia amygdalina* Del.	Asteraceae	Bitter leaf	Shrub	Leaves	[29]
*W. somnifera* L. Dunal	Solanaceae	Indian ginseng	Shrub	Whole plant	—
*X. Americana* Linn.	Olacaceae	Hog plum	Shrub	Stem bark	—
*X. evansii* hutch	Leguminoceae	—	Tree	Stem bark	—
*Xylopia aeithiopica* Dunal	Annonaceae	African black pepper	Tree	Fruits	[21]
*Zanthoxylium zanthoxyloides* Lam.	Rutaceae	Senegal prickly ash	Shrub	Roots	—

**Table 2:** Preliminary phytoconstituents analysis.

			Phytoconstituents							
Plant	Part	Voucher no.	TAN	FLA	ALK	GLY	SAP	COU	TRI	PST
*X. americana*	Stem bark	KNUST/HMI/2021/SB002	+	+	+	+	+	+	+	+
*J. flava*	Leaves	KNUST/HMI/2014/L084	+	+	+	+	−	+	+	+
*J. multifida*	Stem bark	KNUST/HMI/2021/SB001	+	+	+	+	+	+	+	+
*B. tenufolia*	Stem bark	KNUST/HMI/2020/SB016	+	+	+	+	+	−	+	+
*X. evansii*	Stem bark	KNUST/HMI/2021/SB004	+	+	+	+	+	+	+	−
*A. klaineana*	Stem bark	KNUST/AK1/2013/S005	+	+	+	+	+	+	+	−
*W. somnifera*	Shrub	KNUST/HMI/2021/S001	+	+	+	+	+	−	+	+
*Z. Zanthoxyliodes*	Root bark	KNUST/HMI/2015/RB010	+	+	+	+	+	+	−	−
*L. Lecardii*	Stem bark	KNUST/HMI/2020/SB017	+	+	+	+	+	+	+	+

(+): positive; (−): negative; TAN: tannins; GLY: glycosides; SAP: saponins; ALK: alkaloids; FLA: flavonoids; COU: coumarins; TRI: triterpenoids; PST: phytosterols.

**Table 3:** LC_50_ of plant extracts in cercaricidal activity screening.

Plant specimen	LC_50_ (after 240 minutes of exposure)
*W. somnifera*	1.29
*B. aegyptiaca*	7.1
*X. evansii*	11.14
*J. multifida*	12.9
*J. flava*	22.9
*A. klaineana*	182.81
*X. americana*	194.98
*L. lecardii*	223.87
*B. tenufolia*	309.03
*Z. zanthoxyloides*	851.94

LC_50_: mid-lethal concentration.

**Table 4:** Minimum lethal concentration of plant extracts in cercaricidal activity screening.

Plant specimen	MLC (*μ*g/mL)	Time (minutes)
*W. somnifera*	31.25	120
*B. aegyptiaca*	31.25	120
*X. evansii*	31.25	120
*J. multifida*	31.25	120
*J. flava*	31.25	120
*A. klaineana*	500	90
*X. americana*	125	180
*L. lecardii*	500	90
*B. tenufolia*	1,000	90
*Z. zanthoxyloides*	1,000	90

MLC: minimum lethal concentration.

## Data Availability

The raw data/results from experiments used to arrive at the findings of this study are available from the corresponding author upon request. Previous reports that were used to support this study are cited at relevant places within the text as references.

## References

[1] http://www.who.int/wer.

[2] Fenwick A., Jourdan P. (2016). Schistosomiasis elimination by 2020 or 2030?. *International Journal for Parasitology*.

[3] French M. D., Evans D., Fleming F. M. (2018). Schistosomiasis in Africa: improving strategies for long-term and sustainable morbidity control. *PLoS Neglected Tropical Diseases*.

[4] Olorunlana A. (2022). Dancing in a Cycle: Global Health Agenda and Schistosomiasis Control in Africa. *Parasitic Helminths and Zoonoses - From Basic to Applied Research*.

[5] Ross A. G., Olveda R. M., Li Y. (2015). An audacious goal: the elimination of schistosomiasis in our lifetime through mass drug administration. *Lancet*.

[6] Olveda D. U., McManus D. P., Ross A. G. (2016). Mass drug administration and the global control of schistosomiasis. *Current Opinion in Infectious Diseases*.

[7] Doenhoff M. J., Kusel J. R., Coles G. C., Cioli D. (2002). Resistance of *Schistosoma mansoni* to praziquantel: is there a problem?. *Transactions of the Royal Society of Tropical Medicine and Hygiene*.

[8] Danso-Appiah A., De Vlas S. J. (2002). Interpreting low praziquantel cure rates of *Schistosoma mansoni* infections in Senegal. *Trends in Parasitology*.

[9] Ismail M., Botros S., Metwally A. (1999). Resistance to praziquantel: direct evidence from *Schistosoma mansoni* isolated from Egyptian villagers. *The American Journal of Tropical Medicine and Hygiene*.

[10] WHO (2020). *Ending the Neglect to Attain the Sustainable Development Goals: A Road Map for Neglected Tropical Diseases 2021–2030*.

[11] Kukula V. A., MacPherson E. E., Tsey I. H., Stothard J. R., Theobald S., Gyapong M. (2019). A major hurdle in the elimination of urogenital schistosomiasis revealed: identifying key gaps in knowledge and understanding of female genital schistosomiasis within communities and local health workers. *PLoS Neglected Tropical Diseases*.

[12] Boateng E. M., Dvorak J., Ayi I., Chanova M. (2023). A literature review of schistosomiasis in Ghana: a reference for bridging the research and control gap. *Transactions of the Royal Society of Tropical Medicine and Hygiene*.

[13] Kulinkina A. V., Kosinski K. C., Adjei M. N. (2019). Contextualizing *Schistosoma haematobium* transmission in Ghana: assessment of diagnostic techniques and individual and community water-related risk factorsa. *Acta Tropica*.

[14] https://www.who.int/data/preventive-chemotherapy.

[15] Aryeetey M. E., Wagatsuma Y., Yeboah G. (2000). Urinary schistosomiasis in southern Ghana: 1. Prevalence and morbidity assessment in three (defined) rural areas drained by the Densu river. *Parasitology International*.

[16] Tetteh K., Adjei R., Sasu S., Appiah-Kwakye L. (2004). Index of potential contamination: *Schistosoma haematobium* infections in school children in the Ashanti region of Ghana. *East Africa Medical Journal*.

[17] Dassah S., Asiamah G. K., Harun V. (2022). Urogenital schistosomiasis transmission, malaria and anemia among school-age children in northern Ghana. *Heliyon*.

[18] Kankpetinge C., Tekpor D., Dongdem A. Z., Frimpong K., Odonkor S. (2022). Prevalence and risk factors of urinary schistosomiasis among school-aged children in Devego sub-municipal, Ketu north municipality, Volta region, Ghana. *International Journal of Tropical Disease and Health*.

[19] Agyei-Baffour P., Kudolo A., Quansah D. Y., Boateng D. (2017). Integrating herbal medicine into mainstream healthcare in Ghana: clients’ acceptability, perceptions and disclosure of use. *BMC Complementary and Alternative Medicine*.

[20] Danso-Appiah A., De Vlas S., Bosompem K., Habbema J. (2004). Determinants of health-seeking behaviour for schistosomiasis-related symptoms in the context of integrating schistosomiasis control within the regular health services in Ghana. *Tropical Medicine and International Health*.

[21] Twumasi E. B., Akazue P. I., Kyeremeh K. (2020). Antischistosomal, antionchocercal and antitrypanosomal potentials of some Ghanaian traditional medicines and their constituents. *PLoS Neglected Tropical Disease*.

[22] De Castro C. C. B., Dias M. M., de Rezende T. P., Magalhães L. G., Da Silva Filho A. A. (2013). Chapter 8—natural products with activity against Schistosoma species. *Fighting Multidrug Resistance with Herbal Extracts, Essential Oils and Their Components*.

[23] El-Seedi H. R., Khalifa S. A., Mohamed A. H. (2022). Plant extracts and compounds for combating schistosomiasis. *Phytochemistry Reviews*.

[24] Mtemeli F., Ndlovu J., Mugumbate G., Makwikwi T., Shoko R. (2022). Advances in schistosomiasis drug discovery based on natural products. *All Life*.

[25] Ndegwa F. K., Kondam C., Aboagye S. Y. (2022). Traditional Kenyan herbal medicine: exploring natural products’ therapeutics against schistosomiasis. *Journal of Helminthology*.

[26] Zhang X. G., Li G. X., Zhao S. S., Xu F. L., Wang Y. H., Wang W. (2014). A review of dihydroartemisinin as another gift from traditional Chinese medicine not only for malaria control but also for schistosomiasis control. *Parasitology Research*.

[27] Sheir Z., Nasr A. A., Massoud A. (2001). A safe, effective, herbal antischistosomal therapy derived from myrrh. *The American Journal of Tropical Medicine and Hygiene*.

[28] Asante-Kwatia E., Mensah A. Y., Gyimah L., Forkuo A. D. (2021). The Ghanaian flora as a potential source of anthelmintic and anti-schistosomal agents. *Natural Medicinal Plants*.

[29] Acheampong D. O., Owusu-Adzorah N., Armah F. A. (2020). Ethnopharmacological evaluation of schistosomicidal and cercaricidal activities of some selected medicinal plants from Ghana. *Tropical Medicine and Health*.

[30] Armah F. A., Amoani B., Henneh I. T. (2019). In vitro cercaricidal activity of fractions and isolated compounds of *Erythrophleum ivorense* (Fabaceae) root bark against *Schistosoma haematobium*. *International Journal of Tropical Disease and Health*.

[31] Agyemang-Duah W., Peprah C., Arthur-Holmes F. (2019). Prevalence and patterns of health care use among poor older people under the livelihood empowerment against poverty program in the Atwima Nwabiagya District of Ghana. *Gerontology and Geriatric Medicine*.

[32] Forkuo E. K., Biney E., Harris E., Quaye-Ballard J. A. (2021). The impact of land use and land cover changes on socioeconomic factors and livelihood in the Atwima Nwabiagya district of the Ashanti region, Ghana. *Environmental Challenges*.

[33] Evans W. C. (2009). *Trease and Evans’ Pharmacognosy E-Book*.

[34] Abe E. M., Guan W., Guo Y. H. (2018). Differentiating snail intermediate hosts of *Schistosoma spp*. using molecular approaches: fundamental to successful integrated control mechanism in Africa. *Infectious Disease of Poverty*.

[35] Obare B. A., Yole D., Nonoh J., Lwande W. (2016). Evaluation of cercaricidal and miracicidal activity of selected plant extracts agains larval stages of *Schistosoma mansoni*. *Journal of Natural Sciences Research*.

[36] Botros S., Pica-Mattoccia L., William S., El-Lakkani N., Cioli D. (2005). Effect of praziquantel on the immature stages of *Schistosoma haematobium*. *International Journal for Parasitology*.

[37] Vale N., Gouveia M. J., Rinaldi G., Brindley P. J., Gärtner F., Correia da Costa J. M. (2017). Praziquantel for schistosomiasis: single-drug metabolism revisited, mode of action, and resistance. *Antimicrobial Agents and Chemotherapy*.

[38] Molla E., Giday M., Erko B. (2013). Laboratory assessment of the molluscicidal and cercariacidal activities of *Balanites aegyptiaca*. *Asian Pacific Journal of Tropical Biomedicine*.

[39] Isa M. (2021). Laboratory assessment of molluscicidal and cercaricidal activities of *Balanites aegyptiaca* against vectors of schistosomiasis (*Biomphalaria pfeifferi*). *International Journal of Research and Review*.

[40] Koko W. S., Abdalla H. S., Galal M., Khalid H. S. (2005). Evaluation of oral therapy on mansonial schistosomiasis using single dose of *Balanites aegyptiaca* fruits and praziquantel. *Fitoterapia*.

[41] Amponsah I. K., Armah F. A., Alake J. (2020). In-vitro anti-cercarial activity of extracts and steroidal alkaloids from the stem bark of *Holarrhena floribunda* (G. Don) Dur. & Schinz. *International Journal of Phytomedicine*.

[42] Tekwu E. M., Bosompem K. M., Anyan W. K. (2017). In vitro assessment of anthelmintic activities of *Rauwolfia vomitoria* (Apocynaceae) stem bark and roots against parasitic stages of *Schistosoma mansoni* and cytotoxic study. *Journal of Parasitology Research*.

[43] Chama M. A., Onyame H. A., Fleischer C. (2020). In vitro activities of crude extracts and triterpenoid constituents of *Dichapetalum crassifolium* Chodat against clinical isolates of *Schistosoma haematobium*. *Heliyon*.

[44] Sparg S., Van Staden J., Jäger A. J. (2000). Efficiency of traditionally used south African plants against schistosomiasis. *Journal of Ethnopharmacology*.

[45] Mølgaard P., Nielsen S. B., Rasmussen D. E., Drummond R. B., Makaza N., Andreassen J. J. (2001). Anthelmintic screening of Zimbabwean plants traditionally used against schistosomiasis. *Journal of Ethnopharmacology*.

[46] Bah S., Diallo D., Dembélé S., Paulsen B. S. (2006). Ethnopharmacological survey of plants used for the treatment of schistosomiasis in Niono District. *Journal of Ethnopharmacology*.

[47] Lahlou M., Berrada R. (2001). Potential of essential oils in schistosomiasis control in Morocco. *International Journal of Aromatherapy*.

[48] Kouadio B., Romuald O. E., Georges K. A. (2022). Endogenous knowledge of the Attie people on anti-schistosomiasis medicinal plants in the Adzope Health District Côte d’Ivoire. *ESI Preprints*.

[49] Dar N. J., Hamid A., Ahmad M. (2015). Pharmacologic overview of *Withania somnifera*, the Indian ginseng. *Cellular and Molecular Life Sciences*.

[50] Umadevi M., Rajeswari R., Rahale C. S. (2012). Traditional and medicinal uses of *Withania somnifera*. *The Pharma Innovation*.

[51] Larhsini M., Sebbane R., Kchakech H. (2010). Screening of some Moroccan plant extracts for molluscicidal activity. *Asain Journal of Experimental Biological Sciences*.

[52] Chandrasekaran S., Jalaja V., Gundampati R. K., Sundar S., Maurya R. (2016). Exploring the inhibitory activity of Withaferin-a against Pteridine reductase-1 *of L. donovani*. *Journal of Enzyme Inhibition and Medicinal Chemistry*.

[53] Orabi M. A., Zidan S. A., Sakagami H. (2022). Antileishmanial and lung adenocarcinoma cell toxicity of *Withania somnifera* (Linn.) dunal root and fruit extracts. *Natural Product Research*.

[54] Kushwaha S., Soni V. K., Singh P. K. (2011). *Withania somnifera* chemotypes NMITLI 101R, NMITLI 118R, NMITLI 128R and withaferin a protect *Mastomys coucha* from *Brugia malayi* infection. *Parasite Immunology*.

[55] Devappa R. K., Makkar H. P., Becker K. (2010). Jatropha toxicity- a review. *Journal of Toxicology and Environmental Health, Part B.*.

[56] Da Silva Alves R. R., Rodrigues J. G. M., Teles-Reis A. (2020). Antiparasitic effects of ethanolic extracts of Piper *arboreumandJatropha* gossypiifolialeaves on cercariae and adult worms *of Schistosoma mansoni*. *Parasitology*.

[57] Dos Santos A. F., Fonseca S. A., César F. A., de Azevedo Albuquerque M. C. P., Santana J. V., Santana A. E. G. (2014). A penta-substituted pyridine alkaloid from the rhizome of *Jatropha elliptica* (Pohl) Muell. Arg. Is active against *Schistosoma mansoni* and *Biomphalaria glabrata*. *Parasitology Research*.

[58] Membe F. U., Kadji F. J. B., Boukeng J. H. (2022). In vitro assessment of the cercaricidal activity of *Sida acuta* Burm. F. and *Sida rhombifolia* Linn. (Malvaceae) hydroethanolic extracts, cytotoxicity, and phytochemical studies. *Evidence-based Complementary and Alternative Medicine*.

[59] Abo-Zeid K., Shohayeb M. (2015). Evaluation of the biocidal activity of alkaloids, saponins and volatile oil extracted from *Nigella sativa* seeds against miracidia and cercariae of *Schistosoma mansoni*. *International Journal of Pharmaceutical Science Invention*.

[60] Lyddiard J. R. A., Whitfield P. J., Bartlett A. (2002). Anti-schistosomal bioactivity of isoflavonoids from *Millettia thonningii* (Leguminosae). *Journal of Parasitology*.

[61] Pereira L. P. L. A., Ribeiro E. C. G., Brito M. C. A. (2022). Molluscicidal and cercaricidal activities of the essential oil of *Dysphania ambrosioides* (L.) Mosyakin & Clemants: implications for the control of schistosomiasis. *Acta Tropica*.

[62] Xiao S. H., Keiser J., Chen M. G., Tanner M., Utzinger J. (2010). Research and development of anti-schistosomal drugs in the People’s Republic of China: a 60-year review. *Advances in Parasitology*.

[63] 2010 Population and Housing Census, Ghana (2014). District Analytical Report- Atwiman Nwabiagya District. *Ghana Statistical Service, Accra, Ghana*.

